# The impact of cockroach control intervention on infectious diarrhea in Songjiang District, Shanghai, China - an interrupted time series analysis

**DOI:** 10.3389/fpubh.2025.1646283

**Published:** 2025-08-11

**Authors:** Jie Feng, Chi Zhang, Bowen Pang, Meng Li, Xihong Lv, Ye Yao

**Affiliations:** ^1^Center for Disease Control and Prevention of Songjiang District, Shanghai (Health Supervision Institute of Songjiang District), Shanghai, China; ^2^Institute of Public Health, Fudan University, Shanghai, China

**Keywords:** disease vector, infectious diarrhea, vector control, interrupted time series, effect evaluation

## Abstract

**Introduction:**

Infectious diarrhea, as one of the oldest infectious diseases, has been fought against by people for hundreds of years. From changing lifestyle habits to developing new drugs, people have been tirelessly searching for ways to reduce the burden of diarrhea. This study evaluated the impact of cockroach control intervention on infectious diarrhea from the perspective of public health and explored influencing factors, providing practical suggestions for implementation in various regions.

**Methods:**

2,471 positive cases of diarrhea and 14,788 outpatient visits were included for analysis. Using the annual cockroach control intervention as the interruption time point, we observed the number of cases and visits for 12 months before and after, established interruption time series to evaluate the impact of cockroach control intervention on diarrhea in the population, and used hypothesis test to determine potential influencing factors.

**Results:**

Conducting an annual cockroach control intervention in Shanghai Songjiang District help reduce the number of diarrhea cases by 23.58% and the number of outpatient visits by 10.12% in the following year on average from 2020 to 2022. The temperature during the intervention in June 2022 showed a significant increase compared to 2020 and 2021; The effectiveness of cockroach control interventions was almost entirely reflected in *Blattella germanica*, while *Periplaneta Americana* were not affected.

**Conclusion:**

Under the combination of local natural conditions and comprehensive prevention measures, regular cockroach control interventions have a promising supporting effect on reducing the disease burden of diarrhea in the population and a certain effect on reducing the symptom burden. However, the intervention effect is affected by numerous factors such as temperature and cockroach species. If local baseline data can be consulted before intervention to select targeted intervention time and prevention methods, it is easier to achieve ideal results.

## 1 Introduction

Infectious diarrhea refers to diarrhea caused by various pathogens infecting the intestine, and ranks among the top 10 in the annual health statistics report of the World Health Organization in terms of human mortality burden (1). For example, cholera broke out in Britain and India in the 19th century, and there are still similar reports nowadays in India; Infectious diarrhea is caused by various pathogens, including bacteria, viruses, parasites, etc., and their transmission routes are roughly the same, namely fecal - oral transmission, the direct or indirect contamination of food and drinking water by pathogens excreted by patients or carriers, and then transmitted through ingestion by susceptible populations.

Infectious diarrhea is closely related to contaminated food and water, and some can be transmitted through aerosols of vomit such as norovirus (2). As the main mode of transmission of infectious diarrhea, it is reasonable for people to focus on interpersonal, food and water for prevention and control. But in addition, the indirect transmission effects like mechanical contamination of disease vectors such as cockroaches and flies, are also worth consideration, as they can help spread the bacteria (3). Vector organisms that come into contact with a patient’s feces, vomit, or simple dirt, as well as food raw materials or water sources, can all become mechanical carriers. Researchers have used mathematical models such as Monte Carlo to demonstrate in detail the role of cockroaches as vectors in the transmission of foodborne diseases in human settlements, the effects of cockroach control interventions, and how to more effectively eliminate cockroaches (3), highlighting the potential of vectors in the transmission of foodborne diseases.

People tirelessly search for ways to prevent diseases, and diarrhea is no exception. In recent decades, China has continuously promoted urbanization, rural water and toilet improvement (centralized water supply, flushing toilets), strengthened the management of food and water transportation, actively implemented health propaganda, and developed cholera vaccines. These comprehensive prevention measures have steadily improved the public health service capacity of the whole society (4). Today, Shanghai may not have one suspected cholera case a year, and the incidence rate of infectious diarrhea has been significantly reduced compared to the beginning of this century. As one of the supporting methods, we carry out vector control interventions in our jurisdiction every year to assist cutting off the transmission chain, reducing the possibility of people contracting pathogens and causing diarrhea. Considering that vector intervention has become a routine task, and cockroach control has always been our work focus due to cockroach’s wide distribution, we would like to take cockroach control intervention as an example to evaluate the effect of vector quantity intervention on the burden of diarrhea disease in the population, and explore the factors that lead to disparities in effectiveness, providing practical suggestions for interventions in other regions.

## 2 Materials and methods

### 2.1 Case and intervention

In China, infectious diarrhea is classified into different levels of legally recognized infectious diseases, including Class A “cholera,” Class B “dysentery” and “typhoid fever,” Class C “other infectious diarrhea.” These infectious diseases are regulated by the Infectious Disease Prevention and Control Law to be diagnosed as confirmed cases through clinical “gold standard” diagnosis (pathogen found through laboratory testing) and reported online as records for subsequent inquiry and research.

The vector quantity control intervention is carried out annually, and the task is issued layer by layer from the municipal level. Ultimately, the grass-roots administrative department generally hands over the implementation of vector control activities to third-party pest control operations (PCOs), and local CDC conducts an effectiveness evaluation after a period of time. The intervention is continuously carried out throughout Shanghai in the summer, focusing on high-risk environments such as corridors, underground warehouses, garbage rooms, and rainwater and sewage wells. It includes early prevention (breeding ground control, deployment of slow-release insecticides, rodent and insect control propaganda, bait feeding, insecticide spraying, etc.) and consolidation prevention (ultra-low capacity insecticide spraying, cockroach board sticking, instant bait replacement and secondary feeding, etc.). The effect of an ideal intervention can last for several months.

### 2.2 Study subjects

Songjiang District is one of the suburbs of Shanghai, with a total area of over 600 square kilometers, a warm and humid climate, and distinct four seasons. As of 2023, the total population of the district is nearly 2 million, whose GDP is over 174 billion RMB. Its industry and commerce are well developed, as the southwest gateway of Shanghai, Songjiang District has attracted numerous migrant workers from all over the country, with balanced geographical and population representation.

This study collected continuously on-site monitoring data of cockroach density and special density data before and after intervention from the Disease Prevention and Control Center of Songjiang District, Shanghai from 2019 to 2023 (5, 6); And collected and summarized data on the number of outpatient visits to intestinal clinics in various medical institutions in the district from 2019 to 2023; The positive cases of infectious diarrhea from 2019 to 2023 were collected from the Chinese Disease Prevention and Control Information System, and the specific inclusion criteria are: ① According to the Infectious Disease Prevention and Control Law of the People’s Republic of China, cases that medical institutions are required to report from 2019 to 2023; ② Cases whose current address is in the jurisdiction of Songjiang District, Shanghai; ③ Cases were diagnosed as cholera, typhoid/paratyphoid fever, dysentery, and other infectious diarrhea. The exclusion criteria are: ① Misreported cards; ② Deleted cards; ③ Duplicate cards.

In the meantime, 5 years of historical temperature data of Shanghai were collected from the website of the National Meteorological Administration for subsequent analysis.

### 2.3 Statistical analysis

This study used the Segmented Regression Model (SRM) of Interrupted Time Series Analysis to evaluate the effect of cockroach control intervention on the number of positive cases of infectious diarrhea and the number of visits to intestinal clinics (7). Use Durbin-Watson (DW) test to determine whether there is any autocorrelation in time series if necessary (8–10). If it exists, the orcutt iteration method would be used for elimination. Then, take observation values for 1 year before and after the annual cockroach control intervention time point to establish an interruption time series for analysis and evaluation, exploring influencing factors of the intervention through relative hypothesis testing. The statistical analysis software used in this study was R (4.3.1) and R studio (2023.06.0 + 421). Unless otherwise specified, all statistical tests were two-sided tests, and *p* < 0.05 was considered statistically significant.

### 2.4 Pre-experiment

Before conducting the intervention evaluation, we did more work and conducted preliminary experiments to find the relationship between vector density and the number of diarrhea people in Songjiang District. We collected the densities of four common vectors (mosquitoes, flies, cockroaches, and mice), the number of diarrhea cases, and the number of visits to intestinal clinics. Then, using the number of cases and visits as dependent variables, the densities of the four vectors as independent variables, we performed multiple linear regression. The results showed a good linear relationship between the number of cases and cockroach density in Songjiang District (*t* = 5.81, *p* < 0.001, *R*^2^ = 0.43), and a certain linear relationship between the number of visits and cockroach density (*t* = 6.69, *p* < 0.001, *R*^2^ = 0.33), but not with the other three vectors. Therefore, we chose cockroaches to perform further analysis.

## 3 Results

### 3.1 The overview of observations

This study collected monthly data of cockroach density in Songjiang District, Shanghai from 2019 to 2023, covering a total of 60 months. Cockroach density monitoring generally uses adhesive cockroach paper, which is collected and recorded after 12 h of placement.

This study collected 2,492 case cards from the Chinese Disease Prevention and Control Information System from 2019 to 2023 (currently residing in Songjiang District). Among them, 21 cards were deleted due to duplication or other reasons, so a total of 2,471 case cards were included as positive cases of diarrhea. Apart from one card diagnosed with “bacterial dysentery” and one card diagnosed with “paratyphoid fever,” the remaining 2,469 cards were all diagnosed with “other infectious diarrhea.” The number of visits to the intestinal outpatient department was collected by the prevention and health departments of various medical institutions in Songjiang District over the past 5 years, and compiled monthly in our facility, with a total of 14,788 visits.

The number of positive cases and visits for infectious diarrhea are guaranteed by national laws and local regulations, with high completeness and reliability.

### 3.2 The distribution and trend of observations

**Figure 1:**
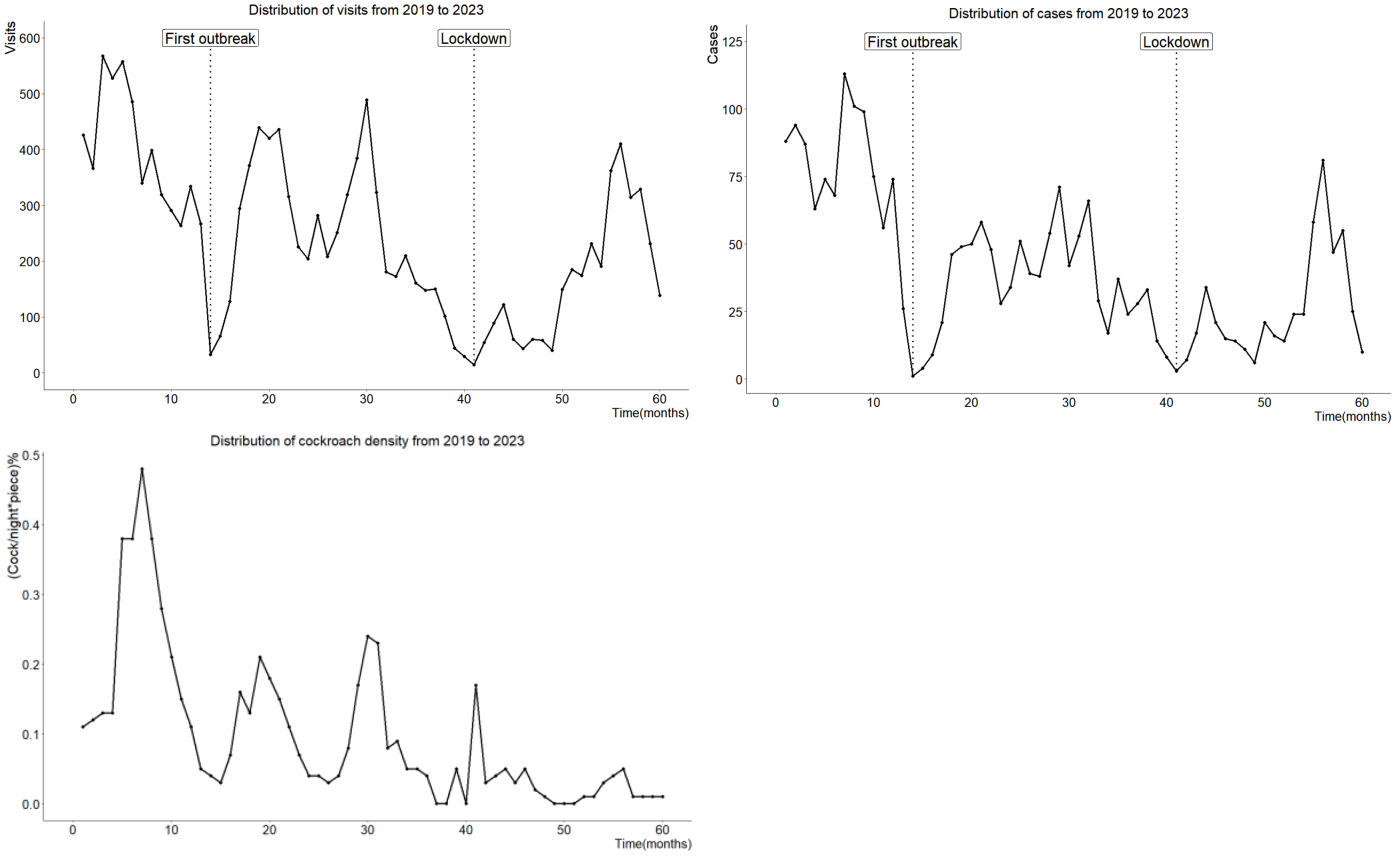
These are distributions of number of diarrhea cases, and number of visits to intestinal clinics and cockroach density from 2019 to 2023. It can be seen that in early 2020 and the first half of 2022, due to Shanghai’s strict implementation of COVID-19 prevention and control, the number of cases and the number of patients appeared two extreme values respectively, which need to be dealt with.

From 2019 to 2023, the density of cockroaches, the number of cases of diarrhea, and the number of visits to intestinal clinics all depicted a similar trend (fluctuation and decrease), as shown in Figure 1.

**Figure 2:**
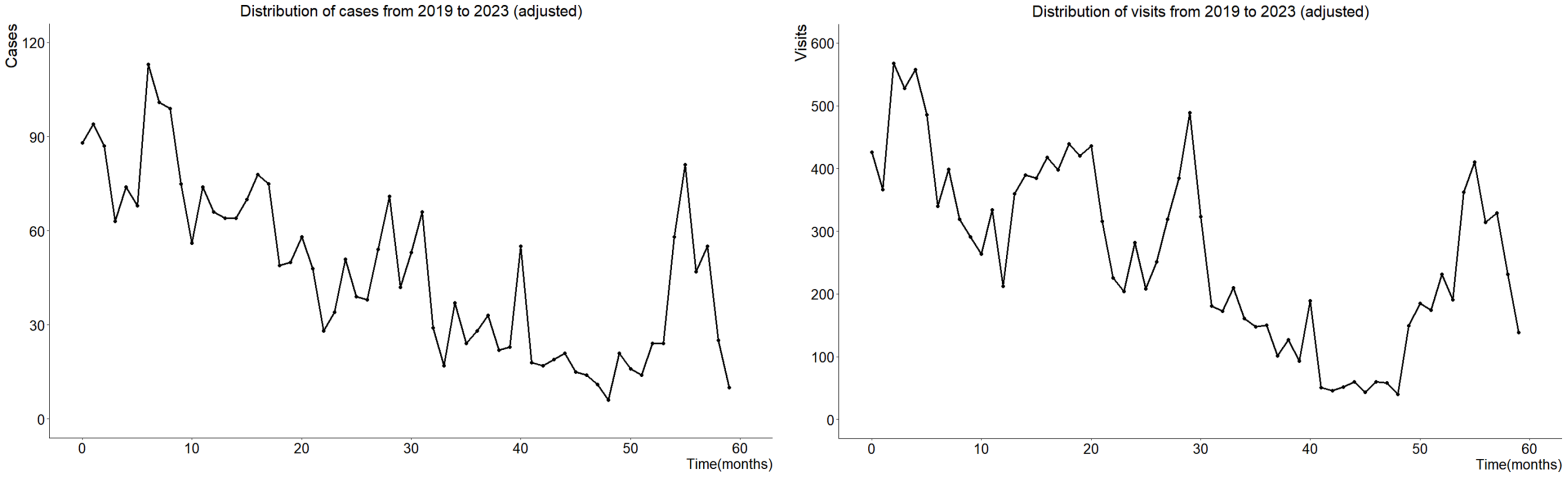
These two pictures show the changes in the number of adjusted diarrhea positive cases and the number of adjusted intestinal outpatient visits over time from left to right. Using raw data from 2019 to 2021 and data from 2021 to 2023, we simulated numbers of cases and patients from January to June 2020, as well as numbers of cases and patients from March to August 2022, respectively.

From 2019 to 2023, 2019, 2021, and 2023 have been relatively less affected by epidemic control measures and can be considered as natural years. Therefore, we used raw data from 2019 to 2021 to simulate the number of cases (visits) from January to June 2020 (6 months), and used data from 2021 to 2023 to simulate the number of cases (visits) from March to August 2022 (6 months), for these two six months have witnessed the strictest epidemic control measures in China. Due to the fact that the simulated data falls within a predetermined time interval, the weighted average regression (Loess smoothing algorithm. Control settings: span: 0.75, degree: 2, family: gaussian, surface: direct, normalize: TRUE) method was used to predict cases for both six-month period. The simulation process was completed by software, and the visualization of data sources and target simulation time periods, as well as the changes in the number of positive cases and visits over time after simulation, can be seen in Figure 2.

### 3.3 Interrupted time series analysis of cockroach control on cases and patients

**Figure 3:**
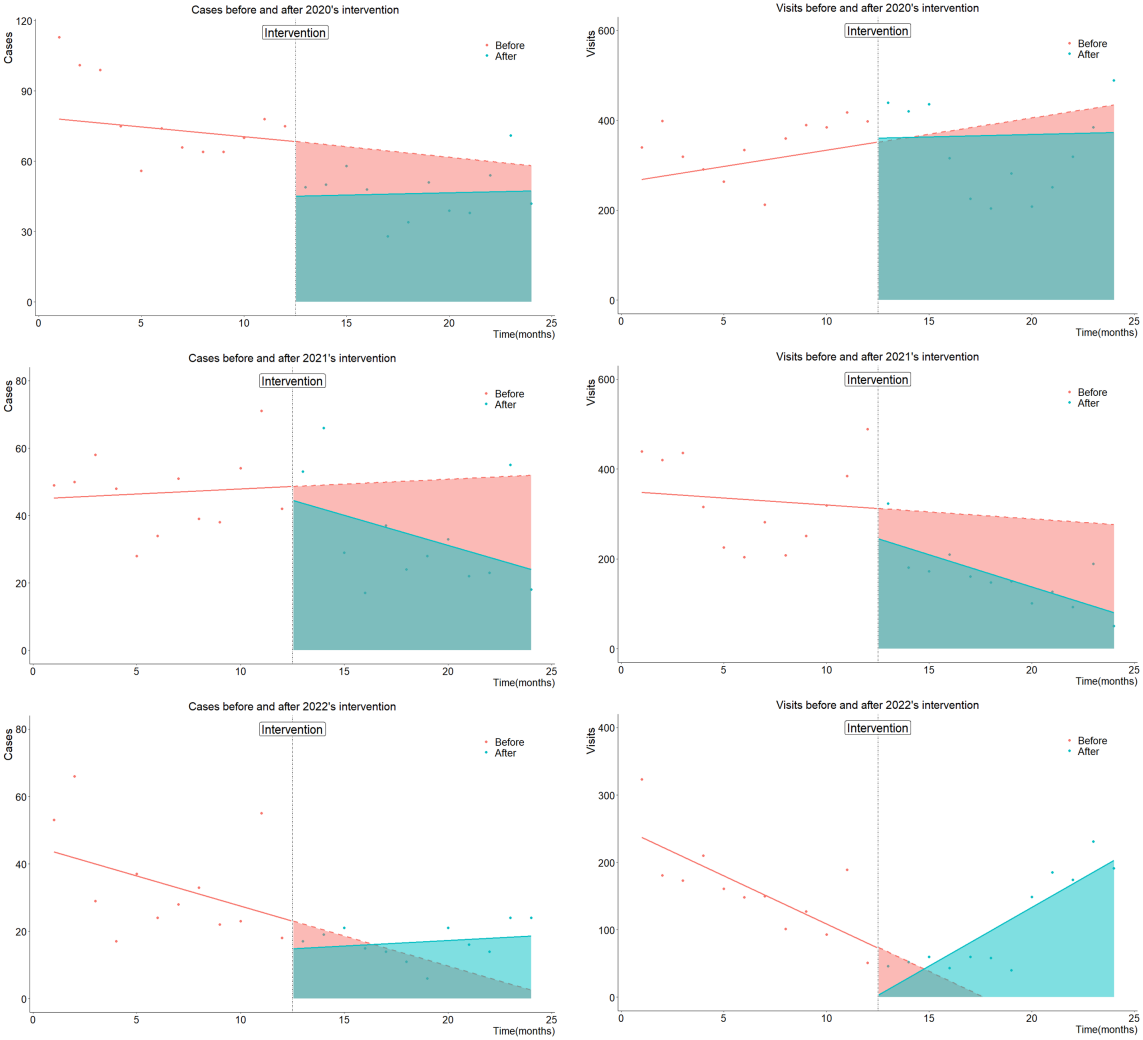
These pictures compare the number of cases of diarrhea and the number of visits to intestinal clinics before and after cockroach control from 2020 to 2022 after adjustment. The left three images show the number of cases, while the right three images show the number of patients seeking medical treatment. Among them, 2020 depicts the iterative regression line, and the remaining 2 years depict regression lines. The larger the difference (difference area) between the red and blue areas, the better the intervention effect.

Data of the past 5 years showed that the specific timing of cockroach eradication varies each year, but they were all between June and early July. For the purpose of comparing the effects, we took the end of June of the natural year as the intervention time point and observe the changes in the number of people before and after a year. Then, an autocorrelation test was conducted, and the results showed that there was a first-order autocorrelation between the number of positive cases and the number of patients seeking medical treatment in 2020, with statistical DW values of 1.150 and 1.115, respectively, and *p*-values of approximately 0.001. There was no autocorrelation in the remaining 2 years. After iteration for 2020, interrupted time series models of 2020, 2021, and 2022 were established, and the graphs and tables were as follows (Figure 3).

As Table 1, the overall effect of cockroach control over the past 3 years has shown a promising trend, with at least two indicators showing positive changes each year, not only in terms of disease burden (cases), but also symptom burden (number of visits). Taking the difference area of cases as an example, the average annual decreased from 2020 to 2022 is 116.39 cases, while the adjusted annual cases of 2020, 2021, and 2022 were 684, 521, and 276 cases, respectively, with an average of approximately 493.67 cases per year. Namely, under the combined effect of natural conditions and other prevention measures, conducting an intervention once a year in Shanghai Songjiang District help reduce the average number of diarrhea cases by 23.58% in the following year from 2020 to 2022. Similarly, after adjustment, the average annual number of medical visits decreased by 282.14 from 2020 to 2022. However, after reducing the impact of COVID-19, the annual number of medical visits in this area in 2020, 2021, and 2022 were 4,204, 3,130, and 1,030, respectively, with an average of 2,788 cases per year. Like the circumstances above, conducting an intervention once a year in Songjiang District help reduce the average number of visits by 10.12% in the following year from 2020 to 2022. From the perspective of preventing and controlling infectious diarrhea and reducing symptoms, carrying out cockroach control interventions every year shows a fairly promising support in Songjiang District.

**Table 1:** Quantitative analysis results of the impact of cockroach control on infectious diarrhea from 2020 to 2022 after adjustment.

Class	Slope change	Mean change	Instantaneous change	Difference area
Cases				
2020	1.056	−31.09	−22.99	202.98
2021	−2.36	−13.08	−4.18	193.14
2022	2.11	−16.92	−8.21	−46.95
Total	/	−61.09	/	349.17
Patients				
2020	−6.139	−11.25	9.11	301.13
2021	−11.15	−172.33	−68.32	1522.80
2022	31.59	−51.50	−70.18	−977.50
Total	/	−235.08	/	846.43

The blue indicator represents the positive change value.

### 3.4 Exploration of influencing factors

It is easy to find disparities in the intervention effect over 3 years from quantitative analysis. This study also explored the influencing factors: first, the impact of COVID-19 prevention and control measures, which is easy to understand. Taking the difference area as an example, the intervention effect showed a trend “2021 > 2020 > 2022,” and 2020 and 2022 were the 2 years when Shanghai’s epidemic prevention and control efforts have significantly increased. In 2020, the first outbreak of COVID-19 transmitting to Shanghai required strengthening control, so did the whole city’s static management from the end of March to the beginning of June in 2022. During these two periods, various measures for cockroach controls, including but not limited to cockroach control points, bait distributions, and site investigations, were more or less restricted. The stricter the control, the more restricted the intervention. Like the diarrhea cases, we calculated the difference before and after the weighted average regression. Without reducing the impact of COVID-19 prevention and control, approximately 683 cases will be passively reduced due to COVID-19 countermeasures in 3 years, with an average of 227 cases per year. The profound impact of this societal change in behavior far exceeded the annual cockroach control. From the perspective of disease prevention, this is a highly efficient method, but it is actually unsustainable.

We also considered climate factors. Although interventions from 2020 to 2022 were carried out in June of the respective year, the specific implementation time was slightly different: the cockroach control in 2020 was gradually carried out in mid June (11th to 20th), the intervention in 2021 was gradually carried out in early June (1st to 10th), and the intervention in late 2022 was gradually carried out in late June (21st to 30th). Using four common temperature indicators (highest temperature average, lowest temperature average, temperature difference average, and average temperature), the temperature characteristics and hypothesis test results of June in Shanghai over the past 3 years were described in Table 2 (Results of ANOVA and Bonferroni tests are redundant to display fully so we extracted those significant).

**Table 2:** Temperature characteristics and hypothesis test results of June in Shanghai from 2020 to 2022.

Type	Highest temperature avg.(°C)	Lowest temperature avg.(°C)	Temperature difference avg.(°C)	Avg temperature (°C)
Description				
June 2020	28.33	22.77	5.57	25.55
Early	27.80	21.30	6.50	24.55
Mid^*^	30.00	23.70	6.30	26.85
Late	27.20	23.30	3.90	25.25
June 2021	28.83	22.07	6.77	25.45
Early^*^	28.20	20.80	7.40	24.50
Mid	29.00	22.30	6.70	25.65
Late	29.30	23.10	6.20	26.20
June 2022	30.87	23.20	7.67	27.03
Early	28.50	20.90	7.60	24.70
Mid	30.20	22.20	8.00	26.20
Late^*^	33.90	26.50	7.40	30.20
Test				
Normality	P_N_ = 0.161	P_N_ = 0.075	P_N_ = 0.108	P_N_ = 0.055
Homogeneity	P_H_ = 0.164	P_H_ = 0.387	P_H_ = 0.734	P_H_ = 0.118
ANOVA^#^	P_J_ = 0.015 P_Y_ = 0.002	P_J_<0.001 P_Y_ = 0.414	P_J_ = 0.040 P_Y_ = 0.001	P_J_<0.001 P_Y_ = 0.014
Bonferroni^#^	P_2022_ < 0.05	P_M_<0.05	P_2022_ < 0.05	P_2022_ = 0.05 P_EJ_ < 0.05

* The time when cockroach control was carried out.

# J stands for disparities between early, mid and late June, Y stands for disparities between 2020, 2021 and 2022, EJ is short for early June.

If we see 2020, 2021, and 2022 as three blocks, and divide each year’s June into three groups: early June, mid June, and late June, we can obtain a sample of 9 groups with 90 days in total. We conducted normality and homogeneity of variance tests on four common temperature indicators. Those who met the normality and homogeneity of variance were subjected to random block ANOVA tests to find statistical differences, and then Bonferroni multiple comparisons were conducted to find specific differences.

The extracted results indicated that all four temperature indicators are considered normal and have equal variances. To be specific, there is a difference in the avg. highest temperature between June 2022 and June of the other 2 years, while there is no difference in the rest. The avg. minimum temperature varies among the early, mid and late June, while there is no difference in the rest. Avg temperature difference shows that there is a difference between June 2022 and June 2020, while there is no difference in the rest. Avg temperature shows that there is a difference between June 2022 and June of the other 2 years, there is also a difference between early and mid to late June, with no difference in the rest.

Finally, the impact of cockroach species. When measuring cockroach density, we also collected cockroach specimens and activities, including cockroaches (both adult and nymph), ootheca, and traces (referring to old ootheca, cockroach feces, and corpses). If we use cockroach density, ootheca density, and positive rate of cockroach traces as indicators and perform paired *t*-tests for each indicator before and after intervention, the results are shown in Table 3.

**Table 3:** Paired *t*-test results of changes in cockroach activities before and after cockroach control.

Year/Type	Before	After	Change	*t* value	*p* value
*Blattella germanica* ^#^					
2020	1.27	1.08	0.19	5.152	0.036
2021	1.28	1.12	0.16		
2022	1.03	0.94	0.09		
*Periplaneta americana* ^$^					
2020	0.20	0.23	−0.03	−2.845	0.105
2021	0.22	0.33	−0.11		
2022	0.21	0.32	−0.11		
Ootheca^&^					
2020	1.59	1.32	0.27	3.890	0.059
2021	1.41	1.02	0.39		
2022	1.20	1.05	0.15		
Trace^*^					
2020	3.99	2.32	1.67	8.690	0.013
2021	3.40	2.17	1.23		
2022	6.03	4.19	1.84		

# The unit of *Blattella germanica* density is one per room, calculated as “the number of *Blattella germanica* divided by the number of positive rooms.”

$ The unit of *Periplaneta americana* density is one per room, calculated as “the number of *Periplaneta americana* divided by the number of positive rooms.”

& The unit of ootheca density is one per room, and the calculation method is “the number of oothecas divided by the number of positive rooms.”

* The unit of cockroach traces is the positive rate, and the calculation method is “the number of positive rooms divided by the number of surveyed rooms.”

In Songjiang District, the distribution of cockroach species is dominated by the *Blattella germanica* and *Periplaneta americana*, and no other cockroach species have been found. As shown in the table above, paired *t*-tests were conducted on the data before and after cockroach control for three consecutive years in 2020, 2021, and 2022. The results showed that there were differences in the density of *Blattella germanica* and the positive rate of cockroach traces before and after the intervention, while there was no difference in the density of *Periplaneta americana* and the density of ootheca before and after the intervention.

## 4 Discussion

Relevant institutes in Songjiang District have achieved the goal of controlling the density of cockroaches by selecting distribution sites, conducting cockroach and insect control propaganda, arranging cockroach bait, and effectively recycling and releasing it for secondary use. This has also helped reduce the burden of infectious diarrhea diseases and symptoms in Songjiang District. In fact, there are relatively consistent reports both domestically and internationally on the positive effects of controlling the number of vector organisms on reducing the disease burden on the population. As early as the beginning of this century, some researchers used the method of community controlled trials to carry out fly control interventions in rural areas of Gambia and Pakistan. They sprayed deltamethrin insecticides on the rural environment of the intervention group within 2 weeks, and continuously monitored the number of flies with traps such as sticky fly papers. At the same time, they asked for records to monitor the incidence of infectious diarrhea in this area. At last, based on the decline in the number of flies, the incidence rate of infectious diarrhea in the intervention group compared with the control group decreased by about 23% (23% in rainy season, 26% in dry season) (11, 12).

Based on the timing and effectiveness of interventions in each year, it can be concluded that in early June each year, when temperatures are still in the early stages of rising (below 26°C), the effectiveness of cockroach control is better than that in mid to late June. As a practical suggestion for cockroach control intervention, it can be used as a reference for regions with similar climates. On a larger scale, in the context of global warming, some areas that were originally unsuitable for biological survival have gradually evolved into suitable living areas, with the invasion of vector organisms; At the same time, the speed of vector reproduction and activity in the original breeding ground have increased (13). Changes in environmental factors such as temperature, precipitation distribution, atmospheric carbon dioxide concentration, and land use and cover are considered key factors in the distribution of vector organisms caused by climate change (14, 15). Studies have shown that rising temperatures lead to changes in the spatial and temporal distribution patterns of vector organisms, expanding their habitats and increasing their numbers, and even driving the invasion of other vector organisms (16, 17), ultimately affecting the effectiveness of vector quantity control interventions. And it is speculated that as the global climate continues to warm, global temperatures will continue to rise (18), and the timing of cockroach control interventions may need to be appropriately moved forward in the future to achieve better intervention effects.

Researches on cockroach species suggest that the current effectiveness of cockroach control interventions for adult cockroaches is mainly reflected in the reduction of *Blattella germanica*, with less impact on *Periplaneta americana*. If cockroach control interventions can be targeted at *Periplaneta americana* in the future, such as replacing insecticides or properly distributing cockroach traps, better intervention effects can be achieved. Like China’s 14th Five Year Plan, environmental management has been placed at the forefront of vector control, followed closely by physical, chemical, and biological control, striving to reduce the living space of vector organisms from the source.

Similarly, researchers in Iran have emphasized the epidemiological significance of controlling cockroach populations. The researchers first described the distribution of different cockroaches in human living environment (widely found in furniture, wall crevices, and even inside electrical appliances), their preferences for water sources, food, and temperature (preferring humidity, with optimal activity between 25°C and 32°C, and a wide range of diets), carrying multiple drug-resistant bacteria and gastrointestinal parasites (such as Bacillus cereus resistant to various common antibiotics such as penicillin and cephalosporin, as well as intestinal amoebas), and their significance as foodborne and iatrogenic infections (19); Then, effective cockroach extermination actions require a combination of bait, traps, insecticides, and even the introduction of nematode parasitic infection of cockroach eggs for biological control. At the same time, the researchers also proposed a series of lethal concentrations (like LT50) in the article to guide cockroach control, and mentioned that under the same concentration of insecticides or bait, larger cockroach species will survive for a longer time than smaller species before dying. This means that larger cockroach species have stronger tolerance, which is consistent with the results of our study (19). Subsequently, the author used a systematic review to describe in detail the various types of pathogens carried by cockroaches, as well as the changes in the pathogen spectrum composed of these pathogens with changes in the temperature of the cockroach’s living environment (20). At the same time, the author also pointed out that cockroaches, as a type of vector organism, possess both importance of disease prevention and challenges ever to do so.

Of course, the limitations of our study are also important. This study focused on exploring some factors that may affect cockroach control interventions, including temperature factors, cockroach species factors, etc. In fact, there can only be more influencing factors in reality. For example, there are other factors in the climate that can be studied, such as humidity, precipitation, and particulate matter; In addition, COVID-19 prevention and control measures and other data are relatively sensitive or already blurry in the local area for some reasons, which is objectively difficult to obtain, to quantify, analyze or publish. Therefore, the deeper impact of COVID-19 can be explored for researchers in other regions; In terms of cockroach species, the main popular species in Songjiang District are *Blattella germanica* and *Periplaneta americana*, and the discussion is also based on these two types of cockroaches. If different cockroach species exist in other regions, the specific effects of cockroach control can also be explored in the future.

## 5 Conclusion

Our study found that under the combination of local natural conditions and comprehensive prevention measures, conducting cockroach control once a year in June help reduce the number of diarrhea cases by about 23.58% and the number of intestinal outpatient visits by about 10.12% on average in the next natural year in Shanghai Songjiang District from 2020 to 2022, reducing the disease and symptom burden of the population. However, the intervention effect is affected by factors such as temperature and cockroach species. If local baseline data can be consulted before intervention to select targeted intervention time and prevention methods, it is easier to achieve ideal results.

## Data Availability

The original contributions presented in the study are included in the article/Supplementary material, further inquiries can be directed to the corresponding author.
