# Supplementary figures and images for: The impact of cockroach control intervention on infectious diarrhea in Songjiang District, Shanghai, China - an interrupted time series analysis
Source: Front Public Health. 2025 Aug 11;13:1646283. doi: 10.3389/fpubh.2025.1646283 (PMC12375580; doi:10.3389/fpubh.2025.1646283)

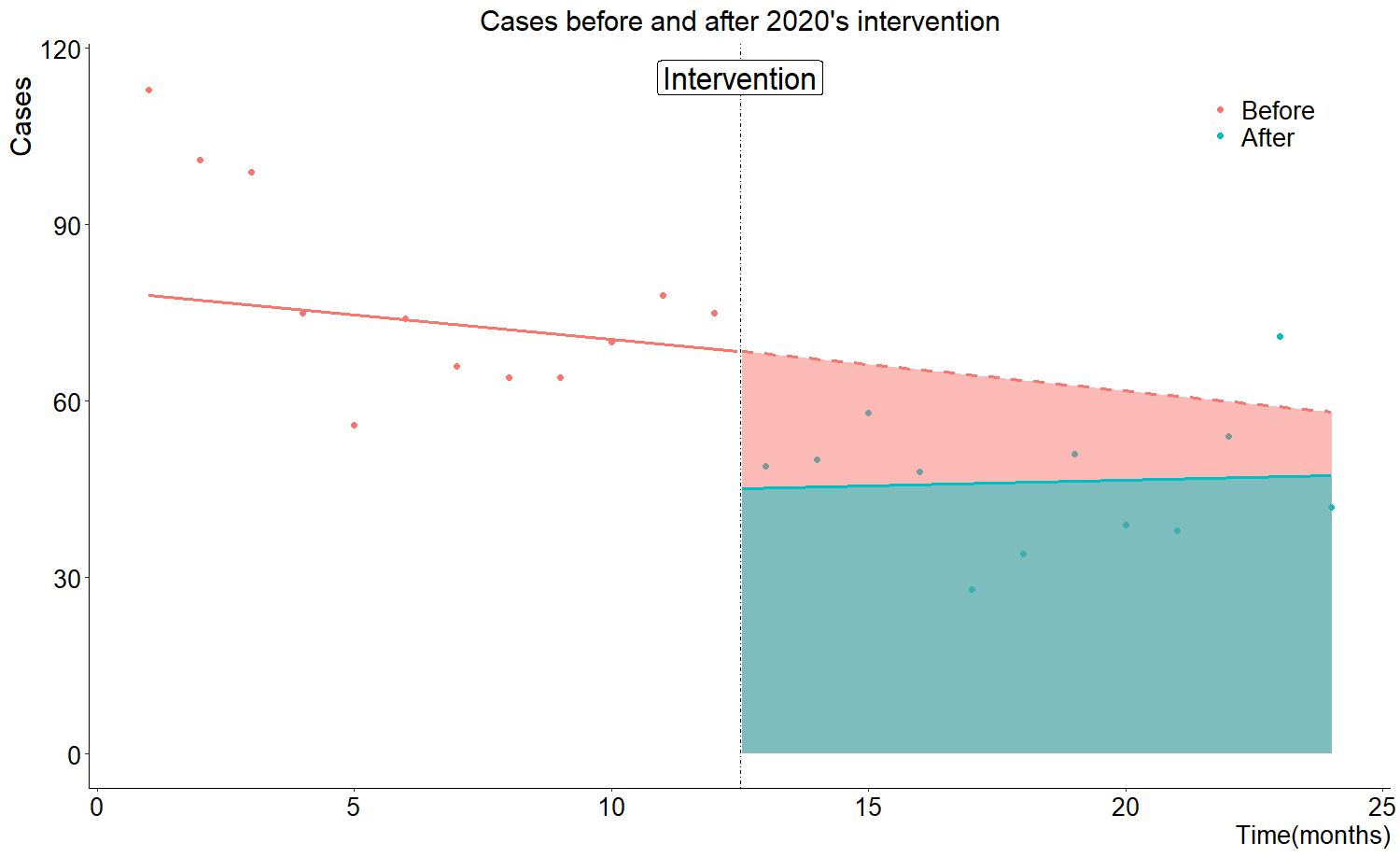

Supplement: Supplementary file 1 [file Data_Sheet_1.ZIP › C2020.png]

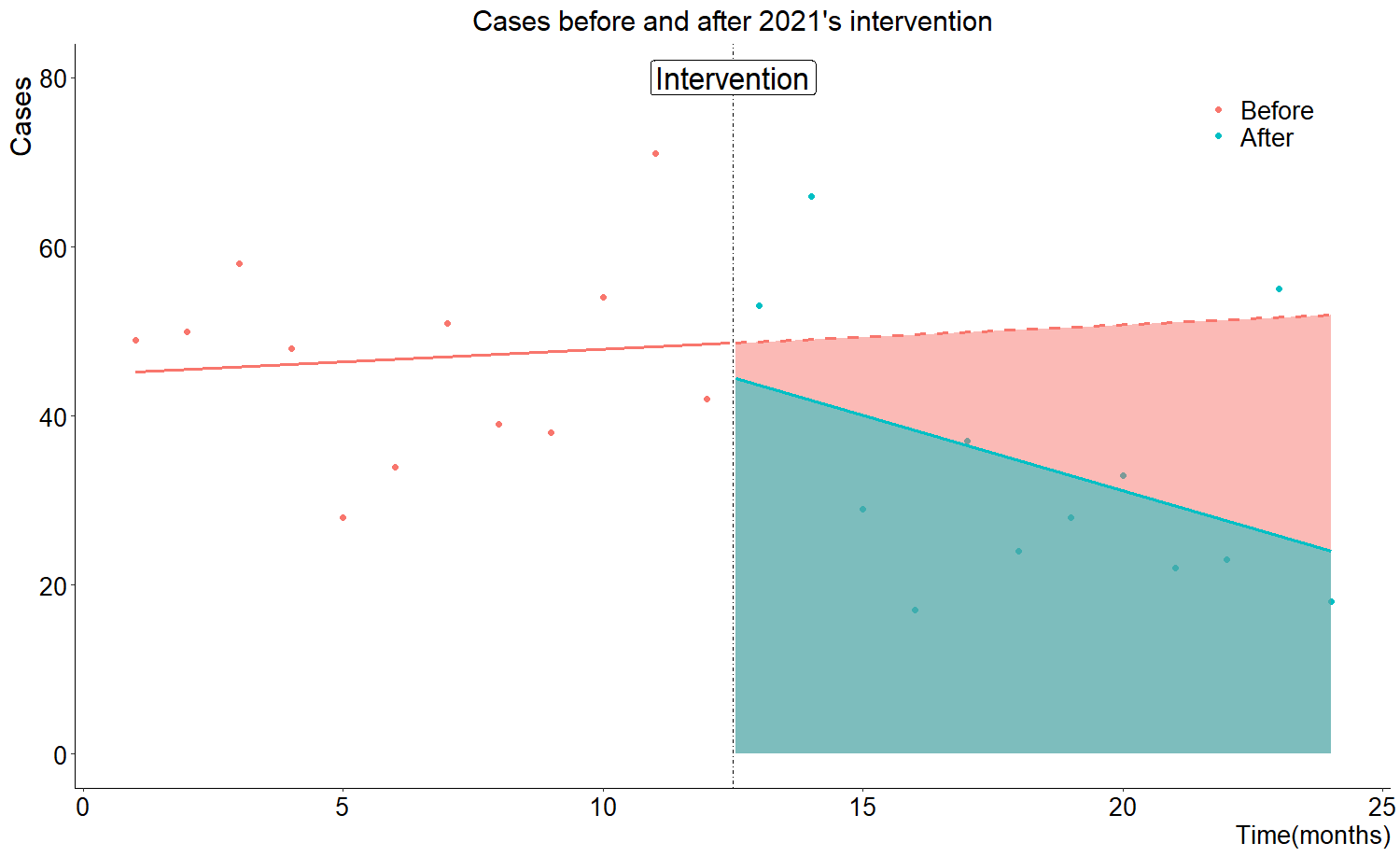

Supplement: Supplementary file 1 [file Data_Sheet_1.ZIP › C2021.png]

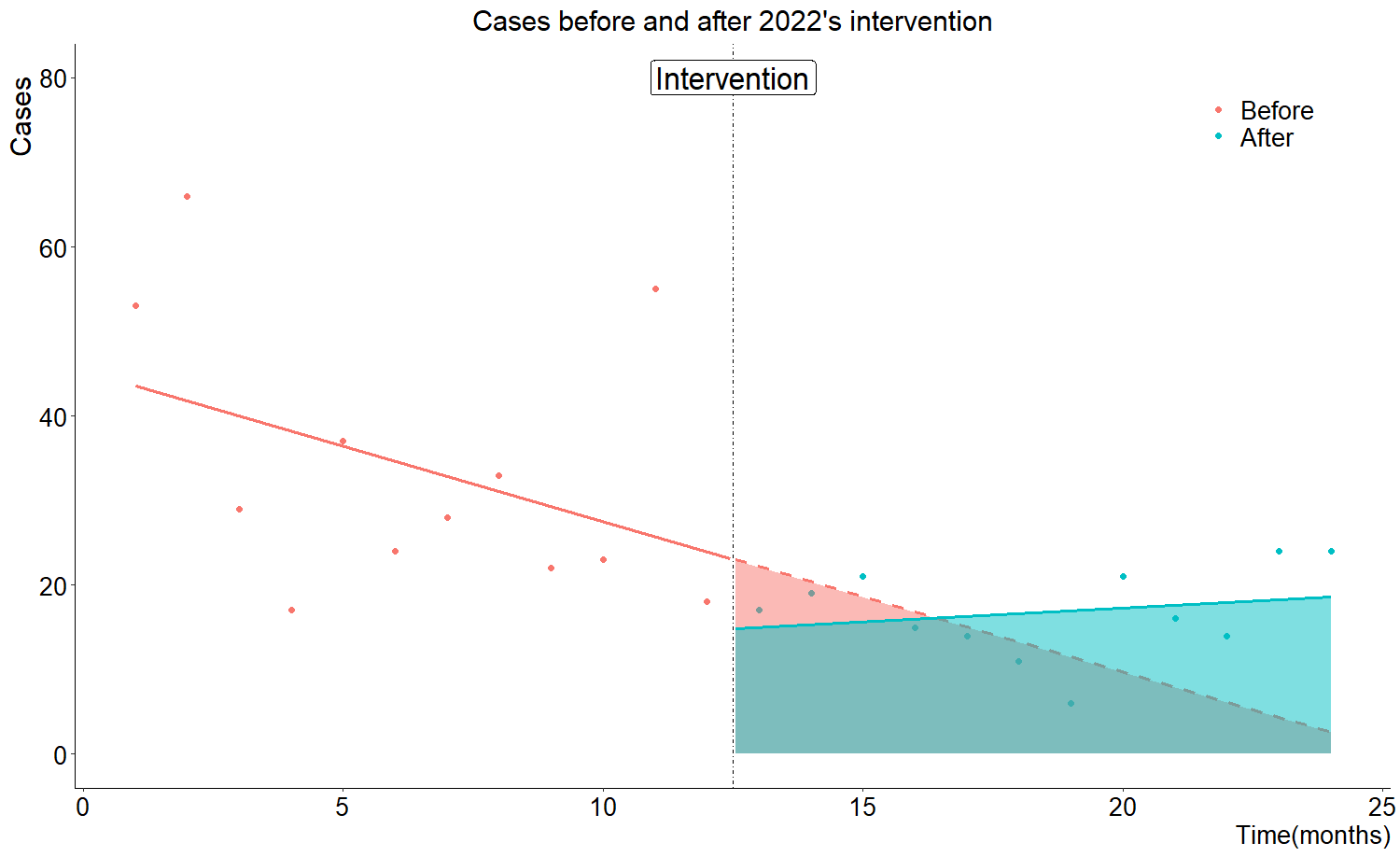

Supplement: Supplementary file 1 [file Data_Sheet_1.ZIP › C2022.png]

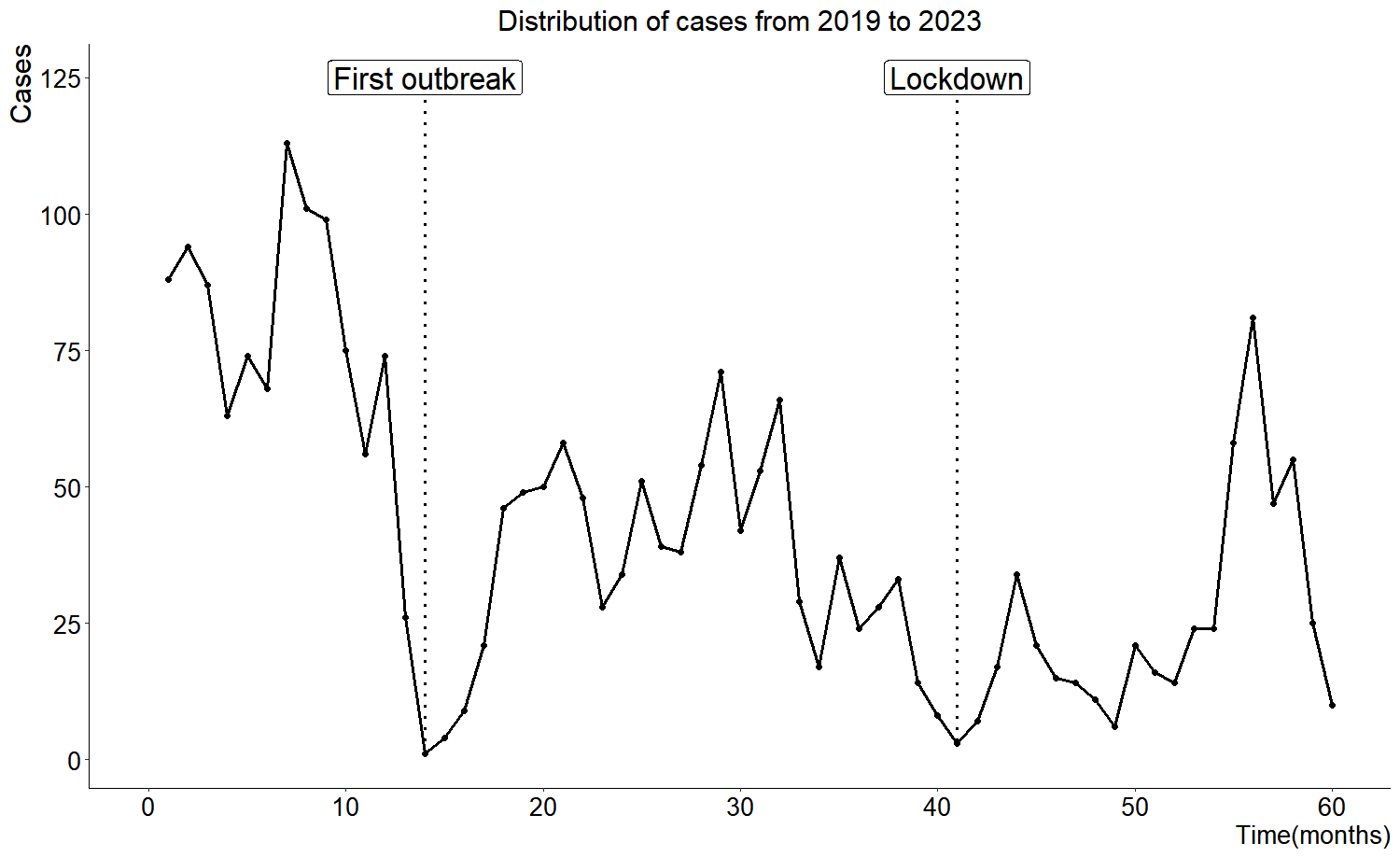

Supplement: Supplementary file 1 [file Data_Sheet_1.ZIP › Cases.png]

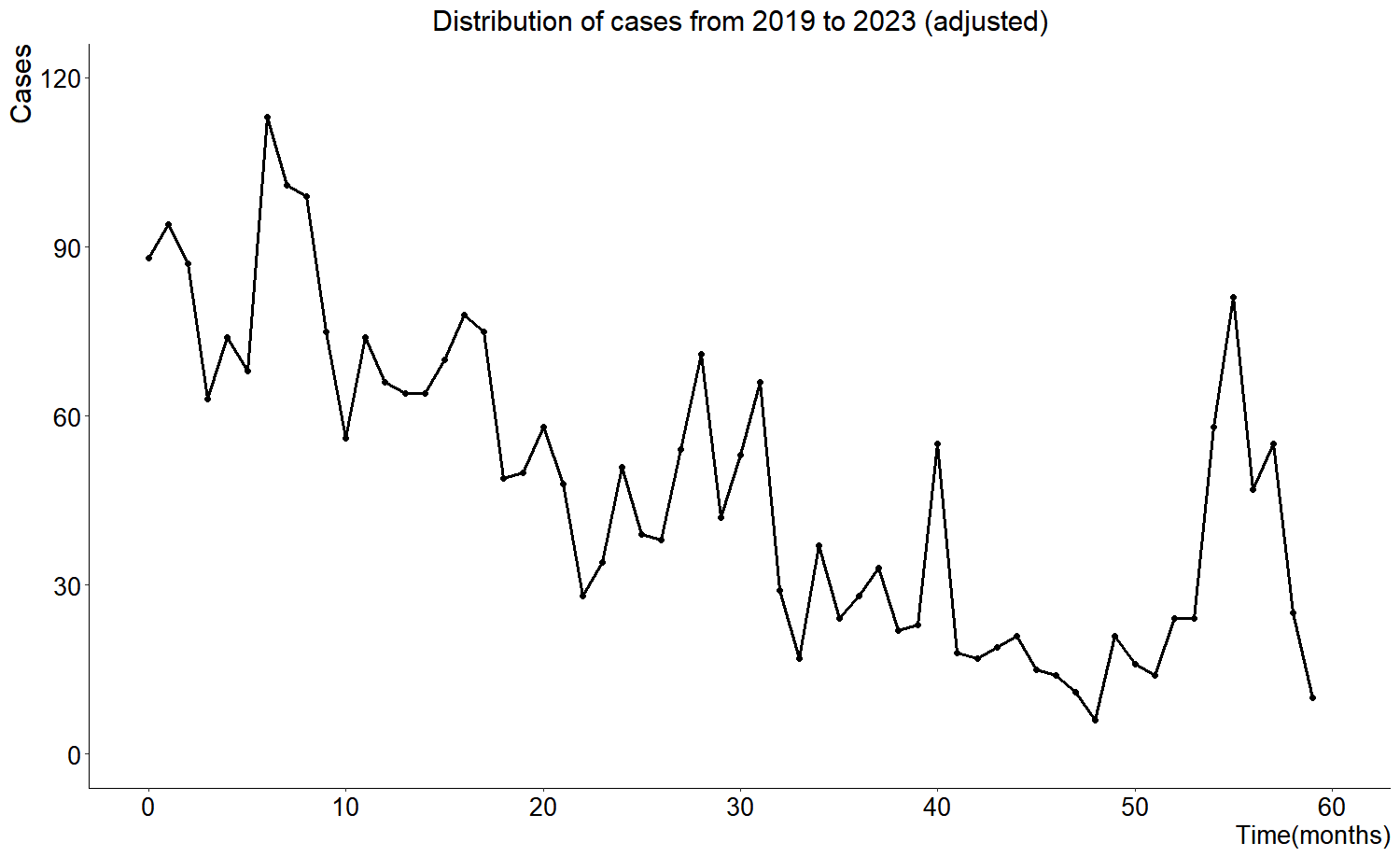

Supplement: Supplementary file 1 [file Data_Sheet_1.ZIP › Casesa.png]

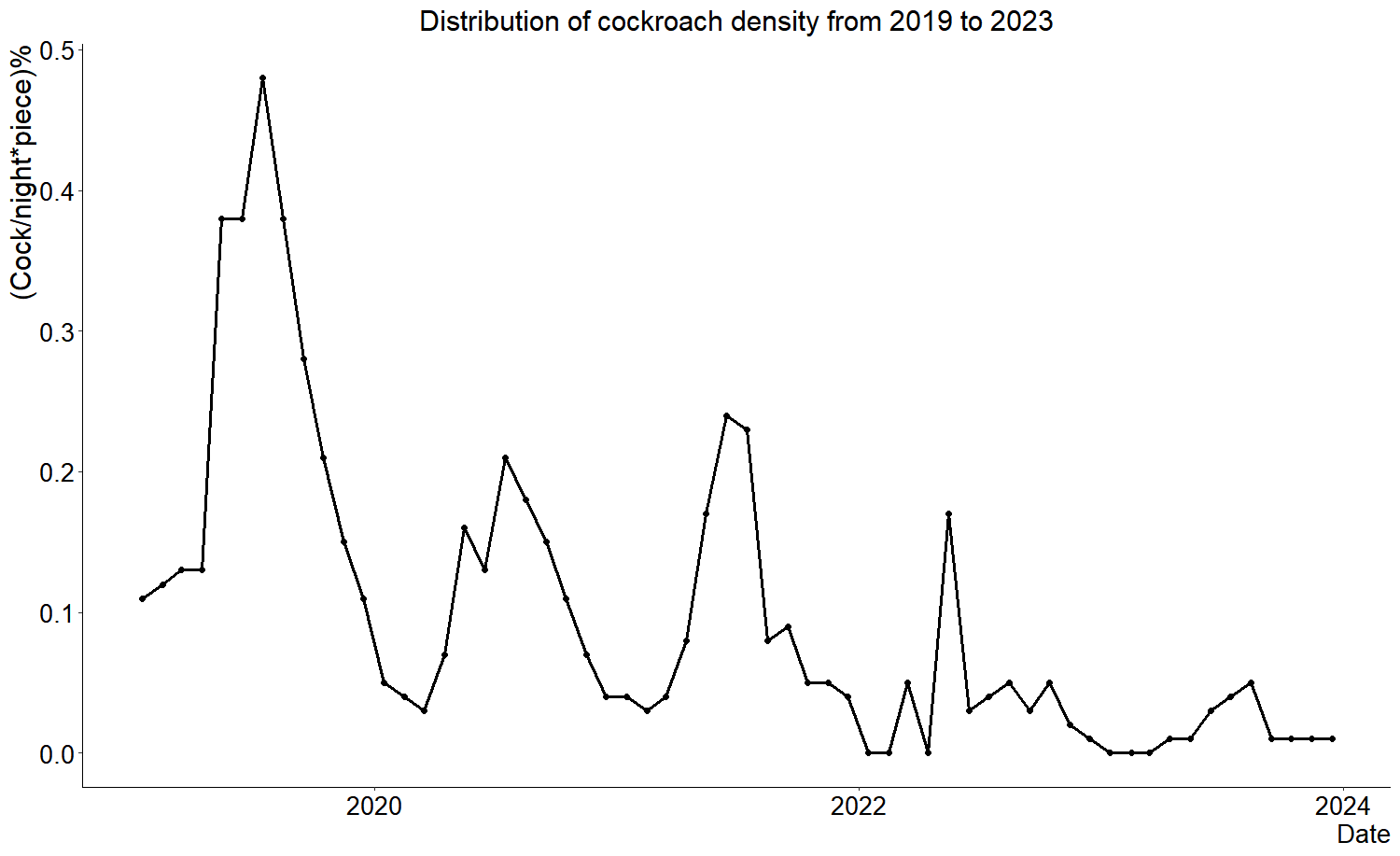

Supplement: Supplementary file 1 [file Data_Sheet_1.ZIP › Cock.png]

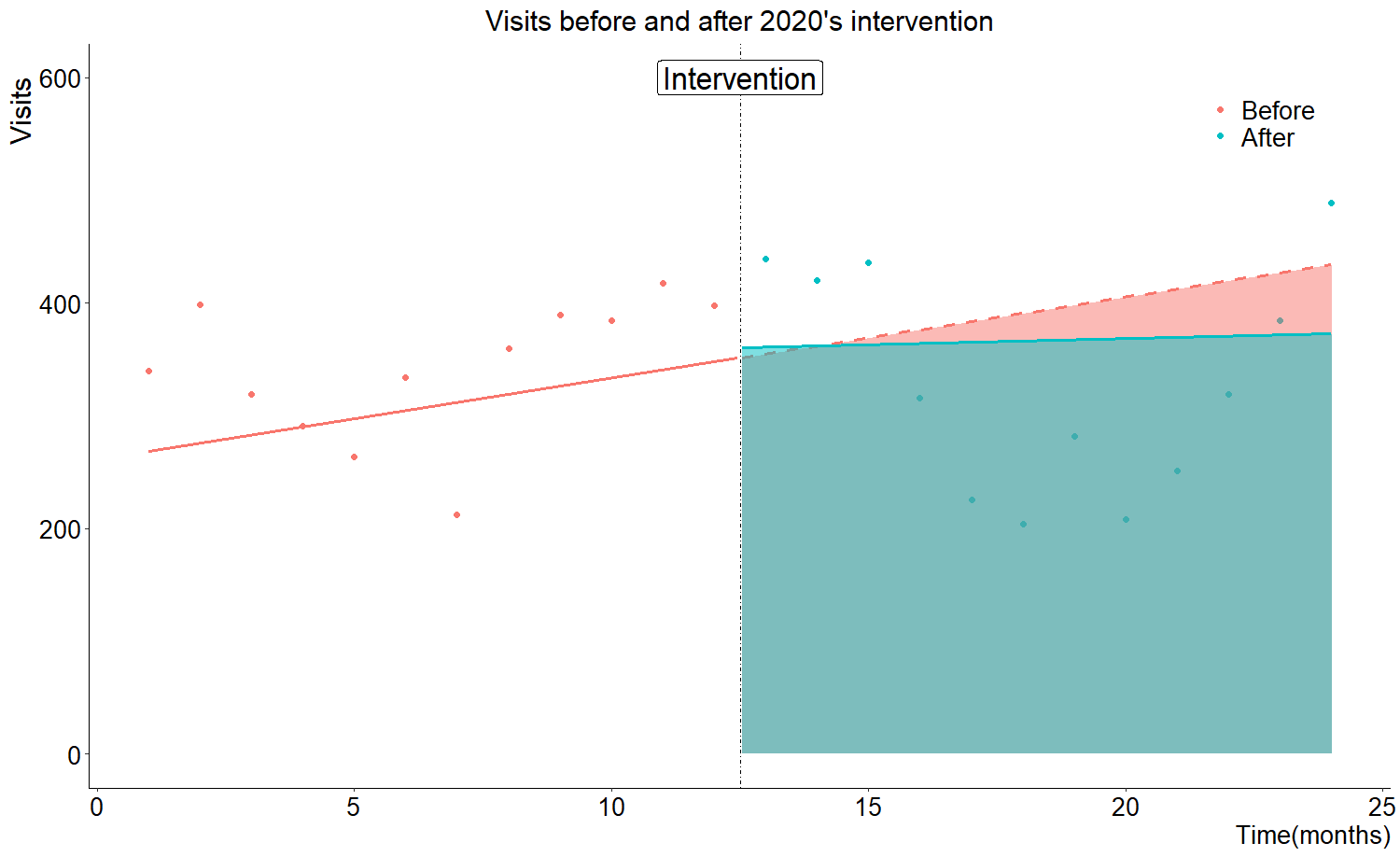

Supplement: Supplementary file 1 [file Data_Sheet_1.ZIP › V2020.png]

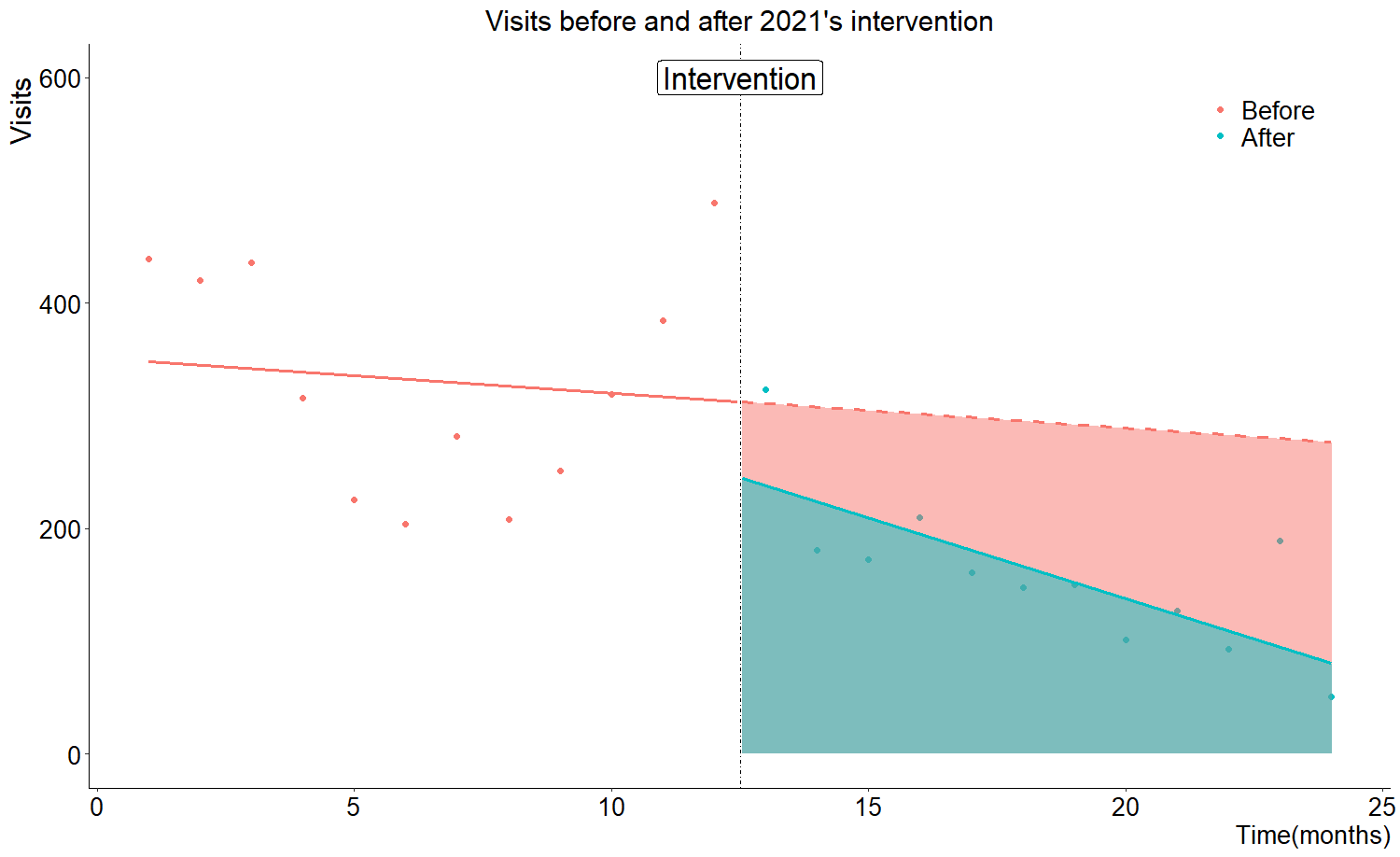

Supplement: Supplementary file 1 [file Data_Sheet_1.ZIP › V2021.png]

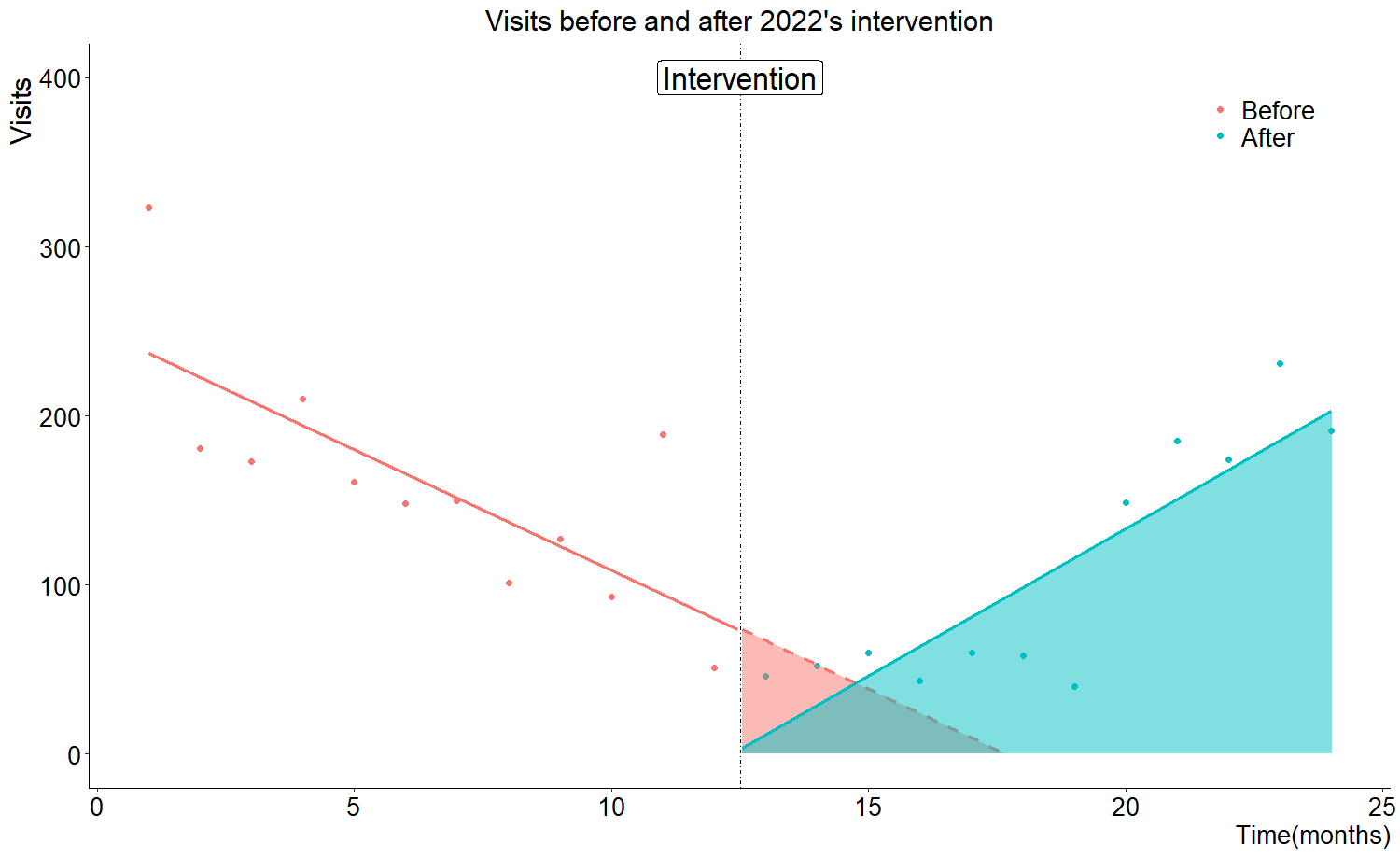

Supplement: Supplementary file 1 [file Data_Sheet_1.ZIP › V2022.png]

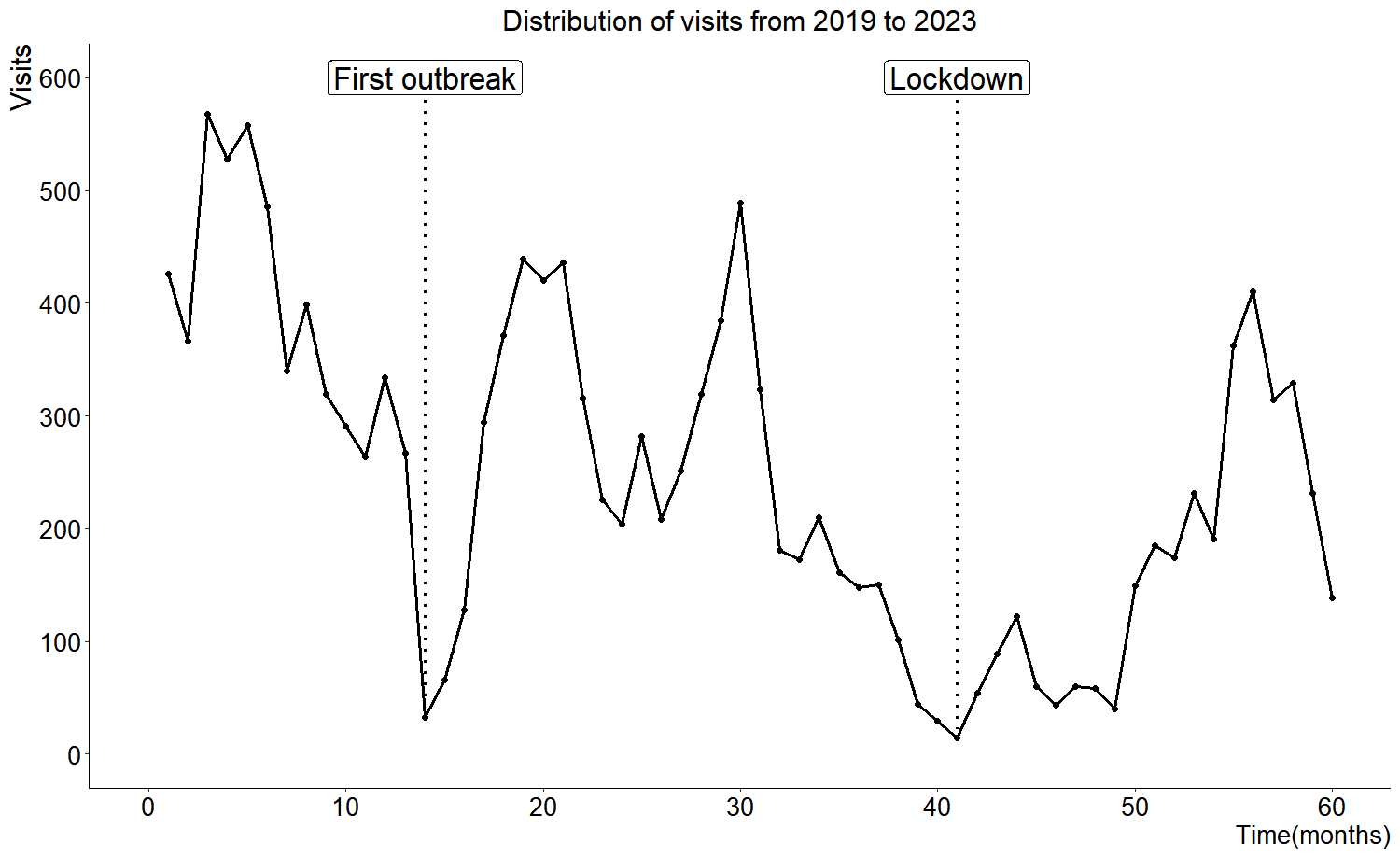

Supplement: Supplementary file 1 [file Data_Sheet_1.ZIP › Visits.png]

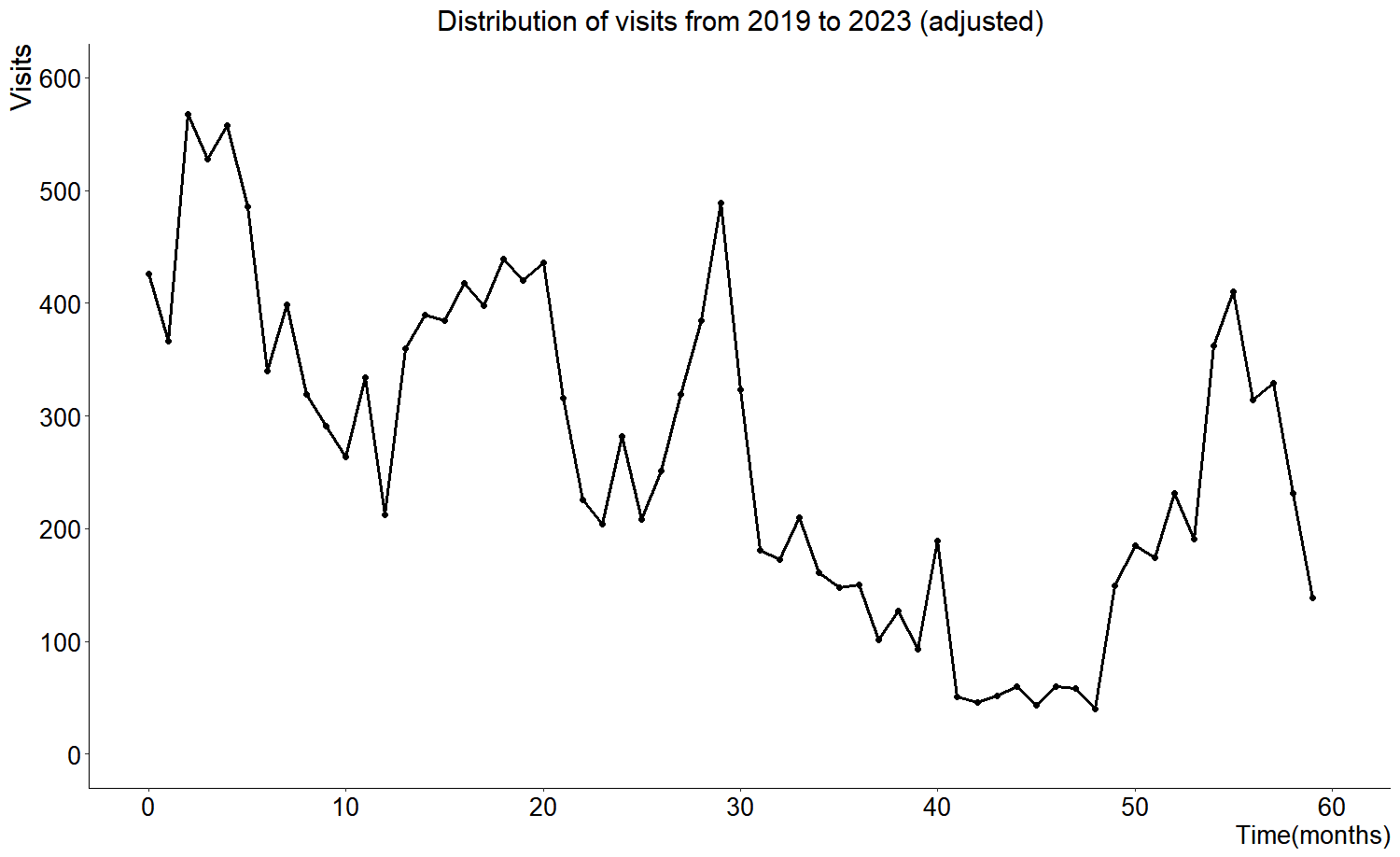

Supplement: Supplementary file 1 [file Data_Sheet_1.ZIP › Visitsa.png]
